# Optimization of Cytokinesis-Block Micronucleus Assay for Clastogenic Risk Assessment of Silver-Containing Nanomaterials

**DOI:** 10.3390/nano16171063

**Published:** 2026-08-26

**Authors:** Ying Yang, Zixuan Shao, Qiu Li, Chenchen Jiang, Yinjing Zhang, Ying Liu, Tun Yuan, Hairuo Wen

**Affiliations:** 1 Hubei Provincial Center for Disease Control and Prevention, National Health Commission Specialty Laboratory of Food Safety, Risk Assessment and Standard Development, Wuhan 430079, China; daisyy1230@163.com (Y.Y.);; 2 Institute of Safety Evaluation, National Institutes for Food and Drug Control, Beijing 100176, China; 3 State Key Laboratory of Drug Regulatory Science, Beijing 102629, China; 4 National Engineering Research Center for Biomaterials, Sichuan University, Chengdu 610064, China; 5 CAS Key Laboratory of Standardization and Measurement for Nanotechnology, National Center for Nanoscience and Technology, Beijing 100190, China; liuy1@nanoctr.cn

**Keywords:** silver nanoparticle, polystyrene nanospheres, in vitro micronucleus assay, nano-silver burn patch

## Abstract

Nanomaterials have been extensively incorporated and are commonly used in everyday life. Rising human exposure to risks has highlighted the carcinogenic hazards from prolonged exposure, prompting the development of “Nanogenotoxicology” as a distinct academic field. However, conventional in vitro micronucleus assay systems cannot ensure adequate contact between nanomaterials and cellular genetic material, which prevents an accurate assessment of the clastogenic risk posed by nanomaterials. In this study, the p53-competent human cell line TK6 and the karyotypically stable conventional cell line CHL were used to conduct a cytokinesis-block micronucleus assay (CBMN), and the cytoskeleton inhibitor cytochalasin B was added to the culture system after nanomaterial exposure. Ag40 (40 nm silver nanoparticles) and polystyrene microspheres were employed as the nanoscale positive and negative controls, respectively. Prior to this study, hyperspectral imaging and inductively coupled plasma mass spectrometry were used to verify that the cellular association of Ag40 was not markedly affected by the addition of rat liver S9 mixture and cytochalasin B. Our results showed that, under exposure periods of 4 h (with or without rat liver S9 mixture), 24 h, 48 h and 72 h, Ag40 at concentrations ranging from 5 to 40 µg/mL significantly increased the micronucleus frequency up to 6%, while polystyrene microspheres showed negative results. Therefore, Ag40 and polystyrene microspheres were identified as the respective nanoscale positive and negative controls for CBMN. The established method was further validated in two independent laboratories using a nano-silver burn wound dressing, and both chemical and nanoscale controls were included. The methodology developed in the present study provided a foundational basis for the development of the standard YY/T 1897-2023 in China.

## 1. Introduction

Nanomaterials have been extensively incorporated into daily products and are now commonly used in everyday life. Nanotechnology is driving profound transformations and achieving breakthrough advancements in food packaging, high-end medical device manufacturing, medical imaging diagnostics, and pharmaceutical research and development. As the risk of human exposure to nanomaterials increases, the potential carcinogenic risks associated with long-term exposure have also attracted widespread attention [1]. While biological macromolecular nanomaterials exhibit no apparent genotoxicity risk, inorganic nanomaterials—especially metallic nanomaterials—could penetrate the cell nucleus and persistently accumulate, thereby potentially inducing chromosomal or DNA damage [2,3]. The genotoxic effects of inorganic nanomaterials are thus regarded as a key focus in nanomaterial safety evaluation. At present, genotoxicity assessment is a mandatory requirement prior to the commercialization of nanomaterial-based products, and “Nanogenotoxicology” has consequently emerged as an independent academic discipline [4,5].

The cytokinesis-block micronucleus (CBMN) assay is widely adopted as a standard method for assessing the genotoxicity of pharmaceuticals and medical devices, primarily through the detection of potential chromosomal damage. However, when applied to nanomaterials, conventional CBMN protocols require substantial methodological adaptation to ensure adequate contact between nanomaterials and cellular genetic material [6,7]. Due to their unique nanoscale effects, nanomaterials cannot cross the cell membrane efficiently via passive diffusion like small-molecule drugs. Instead, they must enter cells through energy-dependent endocytosis, a process that relies on dynamic actin cytoskeleton rearrangement. Moreover, they can penetrate the nuclear membrane and directly interact with chromosomes and DNA [2,3]. Additionally, inorganic nanomaterials have high chemical stability and poor biodegradability. Once they enter cells, they are difficult to clear metabolically and tend to accumulate long-term in lysosomes or the nucleus [8]. However, the exposure time used in conventional methods is usually 3–6 h, with the longest being 24 h. Serum in the culture medium, the S9 metabolic activation system, and albumin can promote the formation of a protein corona that inhibits cellular association of nanoparticles, significantly reducing intracellular uptake efficiency [9].

Furthermore, to induce the formation of binucleated cells, the cytoskeletal inhibitor cytochalasin B (Cyto B) is conventionally added to the experimental system to inhibit actin polymerization, which blocks cytokinesis during the M phase of the cell cycle while permitting nuclear division [10,11]. Cyto B binds to the fast-growing end of F-actin and prevents contractile ring formation, thereby arresting cytokinesis while allowing karyokinesis to proceed [10]. Because actin polymerization is also required for receptor-mediated endocytosis and macropinocytosis of nanoparticles, concurrent Cyto B exposure interferes with the cellular internalization machinery [12]. This interference is further complicated by cell type-specific differences in cell cycle kinetics and actin cytoskeleton dynamics. Human lymphoblastoid TK6 cells, which grow in suspension with a doubling time of approximately 16–18 h, and adherent Chinese hamster lung (CHL) fibroblasts, with a doubling time of approximately 12–14 h, exhibit distinct endocytic capacities and differential susceptibility to Cyto B-mediated disruption of nanoparticle uptake [13,14]. Therefore, delaying the addition of cytochalasin B until after nanomaterial exposure is a key method for improving the accuracy of in vitro micronucleus genotoxicity evaluation of nanoparticles in both TK6 and CHL cell systems [6,7].

After an extensive review and analysis of the literature by the OECD, it was found that current testing produces relatively high false-negative results, and the organization concluded that the conventional testing protocol specified in the OECD Test Guideline 487: In Vitro Mammalian Cell Micronucleus Test (OECD TG 487) cannot adequately evaluate the genotoxicity risk of nanoparticles [15]. The OECD Nanomaterials Working Group has published the “Series on Testing and Assessment No. 359: Study Report and Preliminary Guidance on the Adaptation of the In Vitro Micronucleus Assay for the Testing of Manufactured Nanomaterials” [9]. As stipulated in the guidance, nanomaterial micronucleus testing requires full physicochemical characterization and standardized dispersion procedures. To this end, cell exposure should be prolonged to 12–24 h, cytotoxicity strictly limited to 55 ± 5%, and the peak test concentration for non-toxic samples set at 100 µg/mL [16]. It is also suggested that Cyto B should be added after nanomaterial exposure to avoid interference with cellular uptake [6,9]. Nanomaterial negative, nanomaterial positive, and cell-free interference controls must be included to exclude interference caused by the intrinsic properties of nanomaterials. The guidance clearly states that optimizing experimental conditions and improving the contact between nanomaterials and chromosomes is the core focus of protocol optimization [8]. Furthermore, using cells with intact p53 gene function and DNA damage repair capacity—such as the human lymphoblastoid TK6 cell line expressing wild-type p53—can improve the reliability of test data and reduce false-positive risks associated with rodent p53-deficient cell lines [17,18].

A multi-laboratory blinded validation was conducted under OECD Project 4.174 (“Validation of the In Vitro Micronucleus Assay for Engineered Nanomaterials”), with the revised OECD TG 487 anticipated for completion by 2028 [19]. We have been simultaneously optimizing an in vitro micronucleus test suitable for nanomaterials and conducting inter-laboratory validation since 2021 [20]. Firstly, to ensure adequate cellular association of nanoparticles, hyperspectral microscopy combined with inductively coupled plasma mass spectrometry (ICP-MS) was applied to qualitatively and quantitatively detect nano-silver content in cells under specific culture conditions, as these complementary techniques enable direct spectral visualization and elemental quantification of intracellular nanoparticles, respectively [21,22]. Furthermore, nanoscale negative and positive controls are indispensable components for validating the suitability of a test system [9]. Given the widespread presence of silver-containing nanomedical devices in daily life, and the fact that nano-silver can release silver ions to induce oxidative stress and subsequently cause chromosomal damage [20], 40 nm polyvinylpyrrolidone (PVP)-coated spherical silver nanoparticles (Ag40) were determined as the positive control. PVP coating was employed to enhance the colloidal stability and dispersibility of Ag40 in aqueous media, mimicking the surface modification commonly applied to nano-silver in medical devices and ensuring reproducible exposure conditions in vitro. Ag40 is a well-characterized nanomaterial reference standard with confirmed genotoxic potential, uniform particle size (30–50 nm), and good dispersibility after re-dissolution, ensuring stability in the in vitro culture system [23]. The characteristics of the Ag40 standard are confirmed by a standardized synthesis process, multiple technical characterizations, and a strict quality control system [24]. Furthermore, nanoscale polystyrene microspheres are generally recognized as inert nanoscale particles and were used as the negative control in our study [25]. Polystyrene microspheres were selected to validate the specificity of the test system for nanomaterials, as their chemical inertness and absence of genotoxicity enable discrimination between particle-specific interference (e.g., physical adsorption or optical artifacts) and genuine clastogenic activity [9,25].

In this study, we selected two complementary cell lines: the p53-proficient human lymphoblastoid TK6 cell line and the karyotypically stable Chinese hamster lung (CHL) fibroblast cell line. TK6 cells, derived from human B-lymphoblasts, carry wild-type p53, exhibit a stable karyotype (modal chromosome number of 47), and possess normal DNA repair capacity; they have been recommended as a preferred cell line in OECD TG 487 and related guidelines [17]. Recent advances include the development of CYP-expressing TK6 derivatives and animal-product-free culture systems, which significantly enhance their utility for detecting indirect mutagens and improving human-relevance assessment [26,27]. On the other hand, CHL cells, a classic Chinese hamster lung fibroblast line, have been widely accepted by regulatory guidelines such as OECD TG 473 (In Vitro Mammalian Chromosomal Aberration Test) owing to their clear chromosome morphology, low background aberration rate, and extensive validation data in chromosomal structural aberration analysis [28,29]. The rationale for selecting these two cell lines is threefold. First, as a human-derived p53 functional cell line, TK6 can effectively reduce the risk of non-relevant positive results common in rodent cell lines and improve the predictive capacity for human genetic toxicity risk [17,18]. Second, CHL cells serve as a traditional karyotypically stable reference, providing a standardized baseline for chromosomal aberration analysis that ensures compatibility with historical regulatory datasets [28,29]. Third, the differences between these two cell lines in species origin (human vs. hamster), cell type (lymphoblastoid vs. fibroblast), and DNA damage response mechanisms enable the characterization of test substance genotoxicity from multiple perspectives, thereby enhancing the robustness and reliability of the study conclusions [14,15]. Therefore, the combined use of TK6 and CHL cells aligns with the current trend in genetic toxicology toward humanized, high-predictivity models while retaining compatibility with historical regulatory data, offering a more comprehensive scientific basis for genetic toxicity risk assessment of the test substances.

The objectives of the present study were threefold: (i) to establish an optimized in vitro CBMN assay protocol for nanomaterials by systematically evaluating the effects of exposure duration, S9 metabolic activation, and delayed Cyto B addition on nanoparticle cellular association and genotoxic response in TK6 and CHL cells; (ii) to validate the suitability of Ag40 (PVP-coated silver nanoparticles) and polystyrene microspheres as nanoscale positive and negative controls, respectively, for the CBMN assay; and (iii) to apply the optimized method to assess the clastogenic risk of a commercially available nano-silver burn wound dressing through inter-laboratory collaborative validation, thereby providing a scientific basis and practical reference for the regulatory evaluation of silver-containing nanomedical devices. To achieve these aims, we compared the sensitivity and applicability of TK6 and CHL cells for the CBMN assay under various test conditions, including the addition of S9 and Cyto B and extension of treatment time to 72 h. Subsequently, through inter-laboratory collaborative validation between two participating laboratories, we assessed the chromosomal damage risk of Ag40 and the nano-silver burn patch using this optimized method. This study optimized the test method based on its inherent applicability, providing a foundation for the standardization of in vitro chromosomal aberration test methods, and also offered a reference for the selection of cell lines and treatment conditions for other related in vitro genotoxicity assays.

## 2. Materials and Methods

### 2.1. Cells

Both human lymphoblast TK6 cells and hamster lung fibroblast (CHL) cells were purchased from the Cell Resource Center, Shanghai Institutes for Biological Sciences, Chinese Academy of Sciences (Shanghai, China). TK6 cells were cultured in RPMI 1640 complete medium (VivaCell, Shanghai XP Biomed Ltd., Shanghai, China) containing 10% fetal bovine serum (FBS, VivaCell, Shanghai XP Biomed Ltd., Shanghai, China) and 1% penicillin–streptomycin mixture (Solarbio, Beijing Solarbio Science & Technology Co., Ltd., Beijing, China) at 37°C in a CO_2_ incubator (Thermo Fisher Scientific (Waltham, MA, USA), HERAcell VIOS 160i) with 5% CO_2_. CHL cells were cultured in the same medium and conditions, and passaged when the cell confluence reached 80% to 90%. Before the experiment, the cells were cultured for 18–24 h.

### 2.2. Test Articles and Reagents

**Figure 1:**
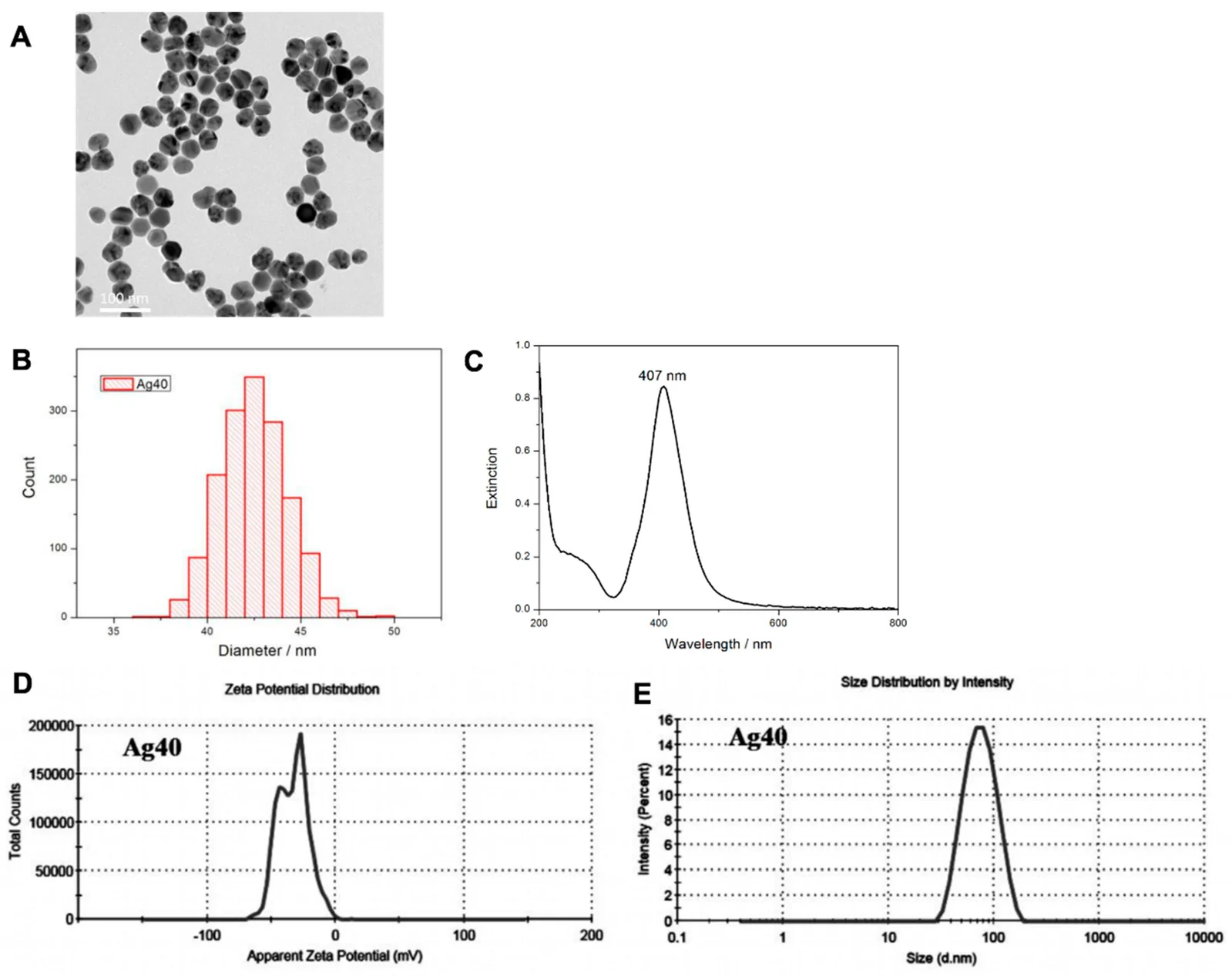
Representative TEM image of nanoparticles. (**A**) Ag40. (**B**) Size distribution of Ag40. (**C**) UV-Vis extinction spectra of Ag40. (**D**) DLS spectrum of zeta potential distribution. (**E**) Size distribution by intensity of Ag40.

Ag40 (Ag40-NM220722) is spherical polyvinylpyrrolidone (PVP)-coated silver nanoparticles, in which PVP serves as a surface stabilizer to enhance colloidal stability and prevent aggregation, provided by the National Center for Nanoscience and Technology (NCNST), Chinese Academy of Sciences (Beijing, China). TEM images (Hitachi (Tokyo, Japan), H-7650) revealed that the size of Ag40 was uniform, and the primary particle size was in the range of 30–50 nm (Figure 1A,B). The extinction spectra of the pristine sample and redispersed powder, recorded using a UV-Vis (Ultraviolet–Visible) spectrophotometer (Shimadzu(Kyoto, Japan), UV-2600), were well matched, demonstrating the absence of aggregation after centrifugation and PVP coating, as well as good redispersibility (Figure 1C). DLS (Dynamic Light Scattering) measurements (Malvern Panalytical (Malvern, UK), Zetasizer Nano-ZS) yielded a zeta potential of −34.2 ± 1.6 mV (Figure 1D) and a hydrodynamic diameter of 67.1 ± 0.5 nm for Ag40 (Figure 1E). Polystyrene nanospheres (JK-05-001-040), used as an inert particulate control to distinguish the biological effects attributable to the physical properties of nanoparticles from those specific to silver, were purchased from Jike Biotechnology (Guangzhou, China). TEM images (Hitachi (Tokyo, Japan), H-7650) revealed that the polystyrene nanospheres exhibited uniform morphology, with particle sizes ranging from 40 to 45 nm. Nano-silver burn patches (0220220421C) were purchased from Anxin Nano Biotechnology (Zhuhai) Co., Ltd. (Zhuhai, China). Extraction solutions were prepared at a ratio of 6 cm^2^/mL in compliance with relevant standards (GB/T 16886.12-2023/ISO 10993-12:2021; GB/T 16886.3-2019/ISO 10993-3:2014) [30,31,32,33]. Samples of nano-silver burn patch dressings were immersed in dimethyl sulfoxide (DMSO) (Solarbio, Beijing, China) and sterile water for injection (Shijiazhuang No.4 Pharmaceutical Co., Ltd., Shijiazhuang, China), followed by extraction with shaking at 37 °C for 72 h in a shaking water bath (EYELA (Tokyo, Japan), NTS-1300). All operations were performed aseptically, and cell seeding procedures were completed on the same day. S9 mixture (metabolic activation system) was purchased from Jiangsu Qishi Biotechnology Co., Ltd. (Jiangyin, China). Cyto B was purchased from Solarbio (Beijing, China). Mitomycin C (MMC) was purchased from TCI (Tokyo, Japan), and cyclophosphamide (CP) was purchased from Sigma-Aldrich (St. Louis, MO, USA). RPMI 1640 medium and FBS were purchased from VivaCell (Shanghai, China), and trypsin was purchased from TransGen Biotech (Beijing, China).

### 2.3. Cellular Association Detection

Cells were treated with Ag40 at concentrations ranging from 2.5 µg/mL to 40 µg/mL under different experimental conditions: 4 h treatment in the presence of rat S9, and 4 h, 24 h, 48 h, and 72 h treatments without S9 metabolic activation. After treatment, cell samples were collected and rinsed with PBS. The localization of Ag40 in cells was determined by measuring the reflection spectra of Ag nanoparticles under a hyperspectral microscope (HSI, CytoViva (Auburn, AL, USA)). The reflection spectra of nanoparticles were used to localize Ag40 and qualitatively assess the cellular association of Ag40.

Cell pellets were sequentially washed three times with ice-cold PBS to remove loosely bound extracellular nanoparticles, followed by overnight pre-digestion in 10 mL concentrated HNO_3_ (65%, metal–oxide semiconductor (MOS) grade) at room temperature, followed by microwave digestion (CEM (Matthews, NC, USA), Mars 6; 120 °C, 5 min; 160 °C, 5 min; 195 °C, 50 min). Digested samples were cooled down and then heated in a heating block (BHW-09C, BOTONYC, Shanghai, China) followed by dilution with 1% (*v*/*v*) HNO_3_ prior to silver quantification by inductively coupled plasma mass spectrometry (ICP-MS; PerkinElmer (Waltham, MA, USA), NexION 300X).

A silver standard stock solution (1000 mg/L as Ag, Certified Reference Material, CRM No. GSB 04-1712-2004, National Center of Analysis and Testing for Nonferrous Metals and Electronic Materials, Beijing, China) was serially diluted with 1% (*v*/*v*) HNO_3_ to prepare calibration standards at concentrations of 0, 1, 2, 5, 10 and 20 µg/L. The calibration curve exhibited excellent linearity over the range of 1–20 µg/L, with a linear regression equation of y = 0.0533x − 0.0227 and a correlation coefficient (R^2^) of 0.9997. Indium (In, 50 µg/L) was employed as an internal standard and introduced online via a peristaltic pump to compensate for matrix suppression and instrumental drift. Radio frequency (RF) power for ICP-MS analysis was 1600 W; plasma gas (Ar) flow rate 18.0 L/min; carrier gas (Ar) flow rate 0.98 L/min; auxiliary flow rate 1.2 mL/min; and monitoring isotope ^107^Ag.

The total silver mass per sample (MAg, pg) was calculated as M*_Ag_* = C × DF × V, where C is the measured concentration (µg/L), DF is the dilution factor, and V is the final volume (L). The number of Ag40 particles (N) was estimated by dividing MAg by the mass of a single spherical Ag40 nanoparticle (d = 40 nm, ρ = 10.5 g/cm^3^):

m*_particle_* = ρ × (4/3) × π × r^3^ = 10.5 × (4/3) × π × (20 × 10^−7^)^3^ ≈ 3.52 × 10^−5^ pg

N = M*_Ag_*/m*_particle_*

Cellular Ag association was expressed as µg Ag per mg cellular protein (BCA assay) to quantitatively determine the cellular association of Ag40 in TK6 and CHL cells.

### 2.4. Cytotoxicity Assay

Cells were seeded in 96-well plates at a seeding density of 2 × 10^5^ cells/mL, followed by exposure to Ag40 at gradient concentrations ranging from 1 to 75 µg/mL for incubation periods of 4 h, 24 h, and 72 h. A broad spectrum of concentrations was prepared, and five wells per treatment were utilized in one treatment period. The cytotoxicity of Ag40 was examined by the adenosine triphosphate (ATP) assay (CellTiter-Glo^®^ 2.0 Assay, Promega), which measures the cellular metabolic activity by quantitating the amount of ATP, an important metabolism parameter in viable cells. The luminescent signals, which reflect the amounts of viable cells, were detected using the VICTOR Multilabel Plate Reader (2030-0050, PerkinElmer), and IC_50_ values were estimated as the concentration of Ag40 for half-maximal viability by Prism 7 (GraphPad Prism 7, San Diego, CA, USA). The viability ratio is calculated using the following equation:

Viability Ratio (%) = RLU_sample_/RLU_vehicle_ × 100%

where RLU is the relative light unit represented as the mean value of four wells, RLU_vehicle_ represents cells not treated with nanorods, and RLU_sample_ represents cells that were treated with different concentrations of Ag40.

### 2.5. Cytokinesis-Block Micronucleus (CBMN) Assay

Cells were exposed to 20 µg/mL polystyrene nanospheres or 5–20 µg/mL Ag40 under multiple regimens: 4 h incubation with S9 metabolic activation, and 4 h, 24 h, 48 h, or 72 h incubation without S9 activation. Post-exposure cells were collected to determine the cytokinesis-block proliferation index (CBPI) and replication index (RI) for the evaluation of cell growth inhibition. Experimental groups included solvent control, nanomaterial negative control, gradient Ag40 treatment groups, and positive control groups (mitomycin C for non-metabolic conditions; cyclophosphamide for S9 metabolic conditions), with three biological replicates set for each group.

For the optimized CBMN assay, cells were seeded in 21 cm^2^ culture dishes (5 mL per dish) at 2 × 10^5^ cells/mL and incubated for 18–24 h to allow complete attachment. Cells were then treated with polystyrene nanospheres, Ag40, or nano-silver burn patch extracts for approximately 4 h, 24 h, and 72 h according to the exposure regimens established above. Following treatment, the culture medium was replaced with fresh medium containing Cyto B at a final concentration of 5 µg/mL, and cells were cultured for an additional two cell-doubling times (24 h). Cells were subsequently harvested, fixed in methanol–glacial acetic acid (3:1, *v*/*v*), spread onto clean slides, air-dried, and stained with Giemsa (Sigma-Aldrich, St. Louis, MO, USA) solution. The grouping settings and biological replications were consistent with those described above.

Morphologically intact binucleated cells were randomly selected for micronucleus scoring. Micronuclei were identified based on the following criteria: (i) staining intensity equivalent to that of the main nucleus; (ii) a diameter ranging from 1/16 to 1/3 of the main nucleus; and (iii) complete spatial separation from the main nucleus [11,12]. Cell debris and nanoparticle aggregates were excluded from analysis. A minimum of 1000 binucleated cells were scored per group to quantify micronucleus-positive cell rates and total micronucleus numbers [13]. Combined with relative cell viability data, statistical analysis was performed. A positive genotoxic result was defined as a significantly elevated micronucleus frequency with a clear dose–response relationship.

Nano-silver burn patches were extracted using DMSO and deionized water, extraction solutions were prepared at a ratio of 6 cm^2^/mL in accordance with GB/T 16886.12-2023/ISO 10993-12:2021 and GB/T 16886.3-2019/ISO 10993-3:2014 [30,31,32,33], and the obtained extracts were serially diluted to relative stock-extract concentrations of 25–100%. These percentage labels (100%, 50%, and 25%) represent relative stock-extract strengths rather than the volume fraction of extract in the final cell culture medium. Based on the reported silver nanoparticle loading of approximately 0.49 g/m^2^ (0.28–0.7 g/m^2^), the theoretical silver concentrations were 294 µg/mL (100%), 147 µg/mL (50%), and 73.5 µg/mL (25%). A constant 200-fold dilution was applied uniformly to all three stock extracts upon addition to the medium to maintain the DMSO concentration at a non-cytotoxic level of 0.5% (*v*/*v*) across all treatment groups, yielding final silver exposure concentrations of 1.47 µg/mL (100%), 0.735 µg/mL (50%), and 0.368 µg/mL (25%). Prior to genotoxicity detection, the cytotoxicity and growth-inhibitory effects of the extracts on TK6 and CHL cells were determined to screen valid test concentrations. The optimized CBMN assay was then performed under unified conditions to assess the chromosomal damage risk induced by nano-silver burn patch extracts.

Inter-laboratory validation was independently conducted at two authoritative institutions: the Institute for Safety Evaluation, National Institutes for Food and Drug Control (Lab A), and the Sichuan Testing Center for Biomaterials and Medical Devices (Lab B).

### 2.6. Statistical Analysis

The data are presented as the mean ± SEM. One-way analysis of variance (ANOVA) was used to test statistical significance of differences among negative control and treated groups, followed by the Dunnett multiple comparison test using SPSS (version 22, IBM, Armonk, NY, USA), and data were considered statistically significant at *p* < 0.05. The figures were prepared using GraphPad Prism 7 for Windows (GraphPad Software, San Diego, CA, USA).

## 3. Results

### 3.1. Cellular Association of Ag40

**Figure 2:**
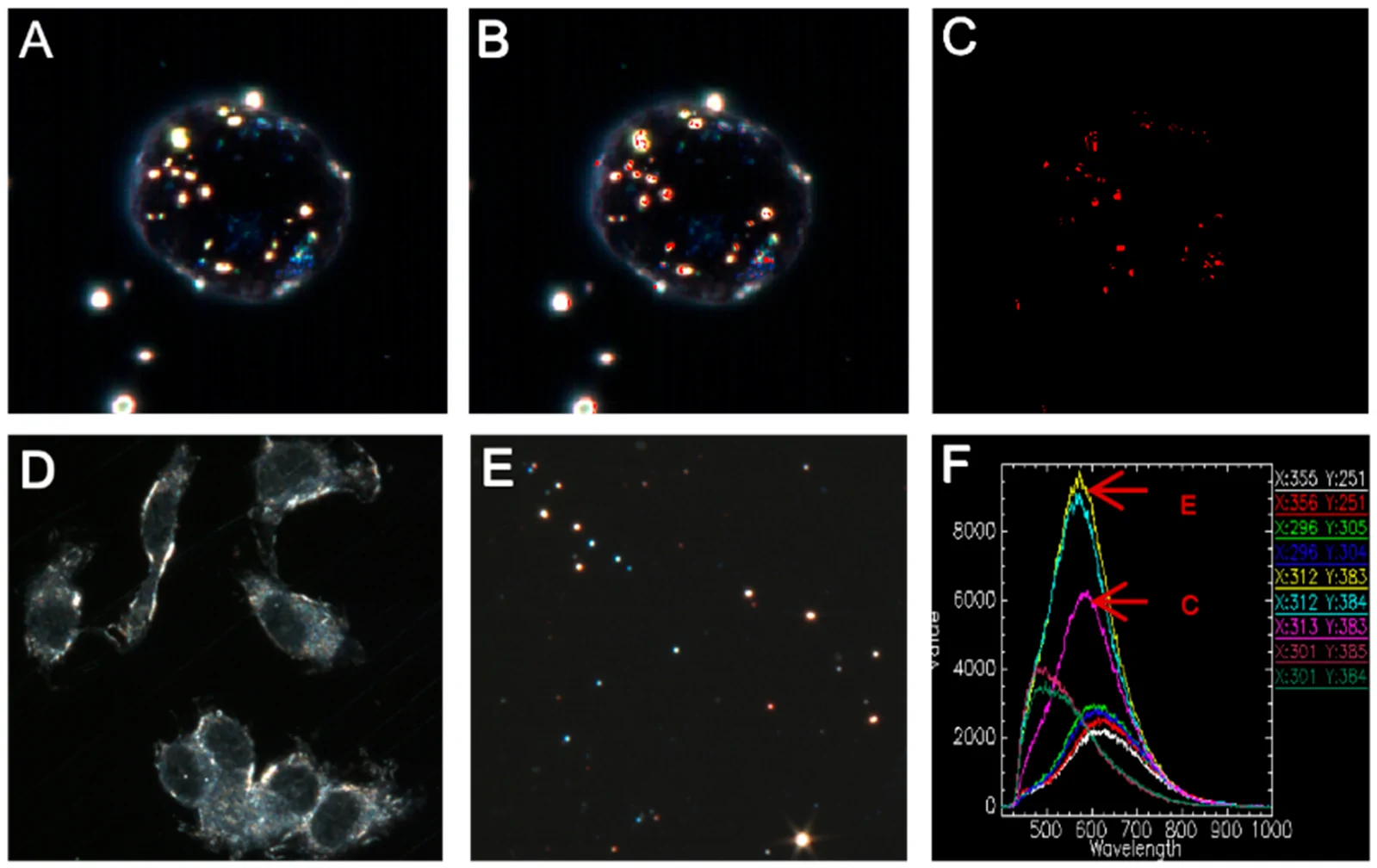
Representative hyperspectral mapping images of cells treated with Ag40. Ag40 was coated with albumin to form the biological corona during cellular uptake. (**A**–**C**) Mapping distribution of Ag40 in CHL. (**D**) Hyperspectral images of CHL. (**E**) Hyperspectral images of Ag40. (**F**) Spectrum diagram of Ag40. The decreased absorption peak intensity observed for intracellularly localized Ag40 (**C**) compared with Ag40 material (**E**) suggests altered optical properties following biomolecular corona formation and endocytosis.

**Figure 3:**
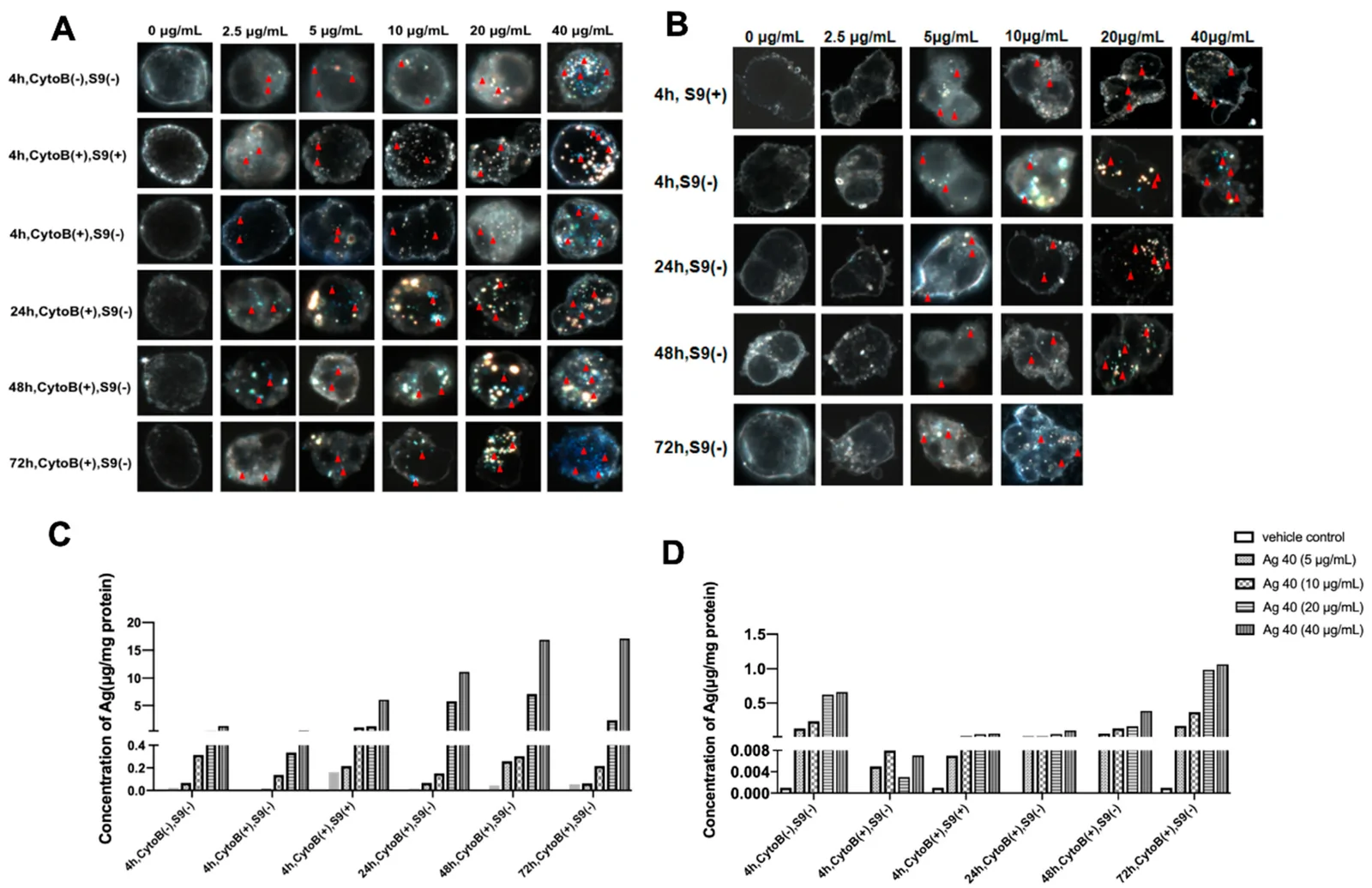
Cellular association of Ag40 in CHL and TK6 cells. Representative mapping images (×100) of Ag40 (red arrow) detected in CHL (**A**) and TK6 (**B**) cells using hyperspectral microscopy are shown after treatment with 0-40 µg/mL Ag40 for 4, 24, 48, or 72 h. Silver content in CHL (**C**) and TK6 (**D**) cell samples, as measured by ICP-MS under identical experimental conditions.

Spherical nano-silver particles were revealed by hyperspectral microscopy in both TK6 and CHL cells; dispersed particles of nano-silver could be observed in both the cytoplasm and the nucleus. The absorption peak of the reflection spectrum of silver nanoparticles was consistent with the reference Ag40 (only the material), suggesting that Ag40 could be taken up and distributed in both cells (Figure 2 and Figure 3A,B).

The intracellular silver content was further quantified using ICP-MS. The silver content in TK6 cells exposed to Ag40 concentrations ranging from 5 to 40 µg/mL was determined to be 0.003–1.063 µg/mg protein, while the silver content in CHL cells exposed to Ag40 ranges from 2.5 to 40 µg/mL was determined to be 0.001–17.12 µg/mg protein, and adherent cultured CHL cells demonstrated a stronger cellular association of Ag40 than suspension-cultured TK6 cells (Figure 3C,D). Under all treatment conditions, the determined silver content was positively correlated with the exposure concentrations and periods. The silver content in cells treated with CytoB was lower than that in cells not treated with CytoB, suggesting that CytoB could inhibit the cellular association of Ag40. In addition, the silver content in cells treated with rat liver S9 mixture was higher than that in cells without S9 mixture treatment. These findings could be attributed to the interaction between chloride ions in the S9 mixture and silver ions from Ag40, forming silver chloride precipitation that adheres to the cell surface [14], making it challenging to fully extract intracellular silver.

### 3.2. Cytotoxicity of Ag40

**Figure 4:**
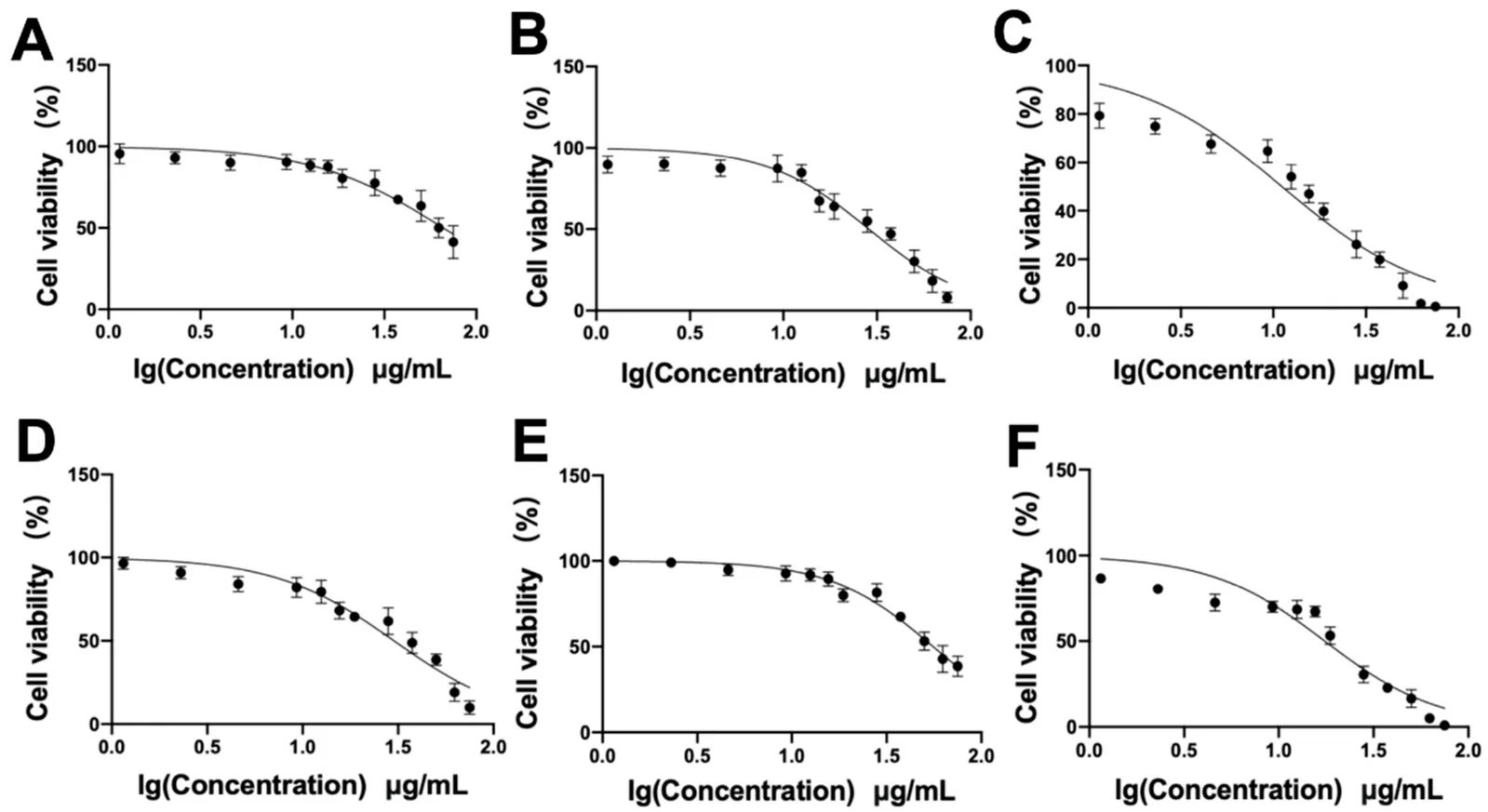
Cytotoxic potential of Ag40 in CHL and TK6 cells. Ag40-induced cytotoxicities in CHL (**A**–**C**) and TK6 (**D**–**F**) subsequent to an exposure period of 4h, 24h or 72h are summarized, respectively.

For cells treated with Ag40 at concentrations ranging from 1 to 75 µg/mL, the IC_50_ values at 4 h, 24 h and 72 h for TK6 cells were 55.69 µg/mL, 30.11 µg/mL and 17.51 µg/mL, respectively; the IC_50_ values at 4 h, 24 h and 72 h for CHL cells were 66.11 µg/mL, 28.63 µg/mL and 11.12 µg/mL (Figure 4), respectively. Under all treatment conditions, the RI% and relative CBPI of polystyrene microsphere (20 µg/mL) and Ag40 (5~20 µg/mL) groups were greater than 45 ± 5% compared with the solvent control group, indicating that these concentrations had no serious inhibitory effect on the growth of TK6 and CHL cells, which could be used for the subsequent CBMN assay.

### 3.3. CBMN Assay for Ag40

**Figure 5:**
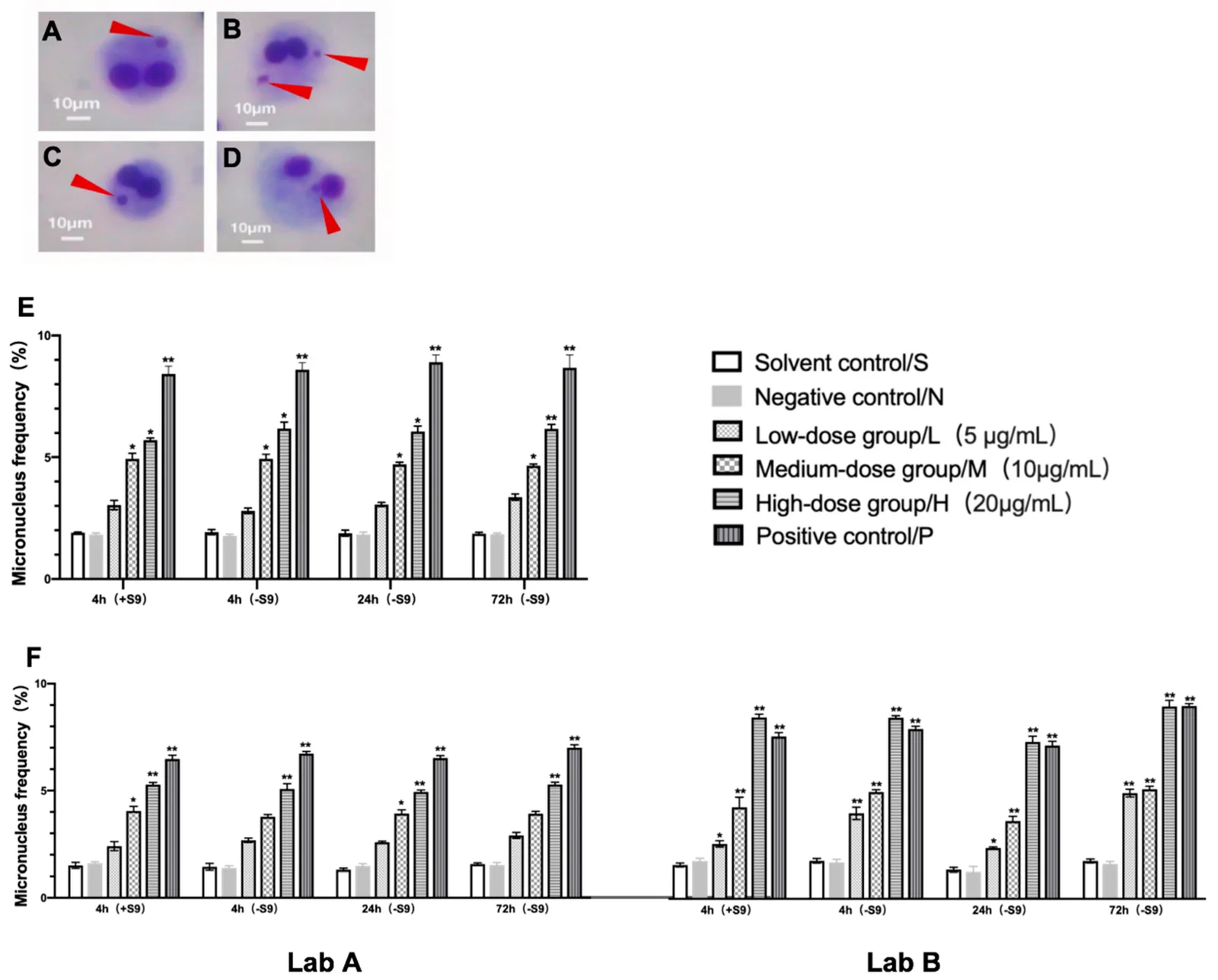
Micronuclei induced by Ag40 under different conditions. Representative images of micronuclei (red arrows) induced by nanomaterials at 20 µg/mL (high-dose group) in CHL (**A**,**B**) and TK6 (**C**,**D**) cells are shown. Micronucleus frequencies induced by nanomaterials in CHL (**E**) cells in Lab A and micronucleus frequencies induced by nanomaterials in TK6 (**F**) cells in both Lab A and Lab B at 5, 10, and 20 µg/mL (low-, medium-, and high-dose groups, respectively) of nano-reference materials. * *p* < 0.05 and ** *p* < 0.01, compared with solvent control group.

Representative images of micronuclei (red arrows) induced by nanomaterials at 20 µg/mL (high-dose group) in CHL (Figure 5A,B) and TK6 (Figure 5C,D) cells are shown. The micronucleus frequency in CHL cells of the positive control was significantly higher than that of the solvent control (*p* < 0.01), verifying the validity of the test system. Increased micronucleus frequencies were observed in medium- and high-dose Ag40 groups in a dose-dependent manner (*p* < 0.05, *p* < 0.01), indicating a positive result. No significant difference was found between the polystyrene nanosphere group and solvent control, which was judged as negative (Figure 5E).

To further verify the applicability of the optimized in vitro CBMN assay for evaluating the genotoxicity of nanomaterials, parallel tests based on Ag40 were conducted in TK6 cells in two independent laboratories (Figure 5F). In Lab A, significantly higher micronucleus frequencies were observed in the medium-dose (M) and high-dose (H) groups following 4 h (+S9) and 24 h (−S9) treatments. In addition, elevated micronucleus frequencies were detected in the H group after 4 h (−S9) and 72 h (−S9) exposure. All these findings were compared with the solvent control (S) group, and the results were accompanied by an obvious concentration-dependent increasing trend. For Lab B, the low-dose (L), M, and H groups exhibited markedly higher binucleated cell micronucleus frequencies than the S group under all experimental conditions, also presenting a positive concentration–response relationship.

Collectively, Ag40 produced a positive outcome in the in vitro CBMN assay, whereas polystyrene nanospheres yielded consistently negative results, with no significant statistical difference in micronucleus frequency observed between the negative control (N) and polystyrene nanosphere (S) groups under all treatment conditions. At concentrations ranging from 5 to 20 µg/mL, this differential response confirms the suitability of Ag40 as a nanomaterial positive control and polystyrene nanospheres as the corresponding negative control in the CBMN assay with TK6 and CHL cells. Notably, the overall micronucleus frequency in CHL cells exceeded that in TK6 cells following Ag40 treatment under identical experimental conditions, consistent with the cellular association analysis demonstrating higher silver accumulation in adherent CHL cells. These findings collectively indicate that the micronucleus frequency induced by silver nanoparticles is positively correlated with cellular exposure levels, thereby validating the established experimental conditions for in vitro micronucleus testing of nanomaterials.

### 3.4. CBMN Assay of Nano-Silver Burn Patch

The survival rates of TK6 and CHL cells treated with nano-silver burn patch extracts at extract concentrations ranging from 3.125% to 100% were not less than 70%. The RI% and relative CBPI values at extract concentrations ranging from 25% to 100% were greater than 45 ± 5% compared with the solvent control group, indicating that these concentrations had no serious inhibitory effect on cell growth, which could be used for the subsequent CBMN test.

**Figure 6:**
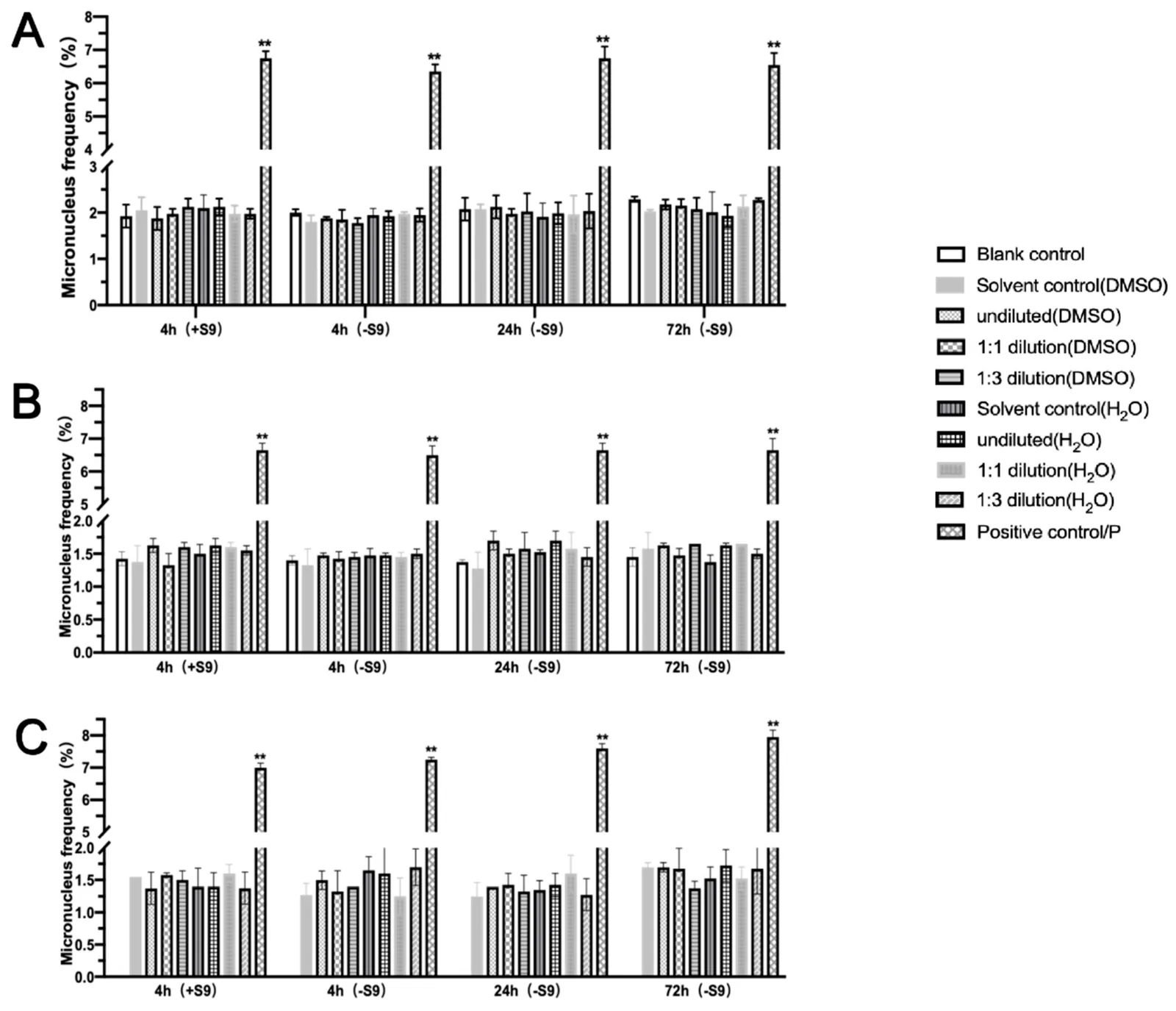
Micronucleus frequencies induced by nano-silver burn patch. The extract of the nano-silver burn patch was tested in a dilution series of the stock solution: undiluted stock (100% concentration), 1:1 dilution (50%), and 1:3 dilution (25%). Control groups included the blank control and the undiluted vehicle control. (**A**) Micronucleus frequencies induced by nano-silver burn patch in CHL cells in Lab A. (**B**) Micronucleus frequencies induced by nano-silver burn patch in TK6 cells in Lab A. (**C**) Micronucleus frequencies induced by nano-silver burn patch in TK6 cells in Lab B. ** *p* < 0.01, compared with blank control group.

Both Lab A and Lab B demonstrated significantly increased micronucleus rates induced by the positive control (*p* < 0.01). The micronucleus rates in cells treated with nano-silver burn patch extracts at extract concentrations ranging from 25% to 100% were not significantly different from those of the solvent control group (*p* > 0.05), and no dose-dependent increase trend was observed (Figure 6). These findings suggested that the nano-silver burn patch had no potential chromosomal damage under the current test conditions. As ICP-MS detection revealed that all silver contents in the cells treated with nano-silver burn patch extracts were lower than the detection limit, the negative result might be due to the low release of silver ions from the nano-silver burn patch.

## 4. Discussion

The in vitro micronucleus assay represents a pivotal component of genotoxicity risk assessment batteries. Nevertheless, conventional CBMN assays were not designed to accommodate the unique physicochemical properties of nanomaterials, presenting substantial limitations in nanogenotoxicity evaluation [34]. In response to these challenges, the present study sought to optimize the CBMN assay with a particular focus on enhancing cellular association for nanomaterial assessment. Given the extensive clinical application of silver nanoparticle-containing products, nano-silver was selected as a nanoscale positive control, and silver-containing nanomaterials were employed to validate the optimized methodology [13].

To facilitate efficient cellular internalization of nanomaterials, several critical parameters were systematically optimized. Sample dispersions were prepared via standardized ultrasonication protocols, cell exposure duration was extended to 12–72 h to ensure coverage of at least one complete cell cycle [14,15], and cytotoxicity was strictly controlled within 55 ± 5% to preclude false-positive outcomes arising from physical membrane damage and cellular debris interference. Conventional assays typically incorporate only chemical negative and positive controls, which are insufficient to verify the applicability of the testing system for nanomaterials [35].

While recent reviews, including the comprehensive analysis by Alund et al. [13], have underscored the general applicability of the micronucleus assay for nanogenotoxicity screening, the present study advances beyond broad methodological recommendations by establishing a systematically optimized CBMN framework characterized by three principal innovations: (i) the incorporation of nanoscale-specific positive (Ag40) and negative (polystyrene nanospheres) controls; (ii) the development of a combined qualitative and quantitative approach integrating hyperspectral imaging (HSI) with inductively coupled plasma mass spectrometry (ICP-MS) [36] to rigorously verify cellular uptake efficiency and intracellular localization; and (iii) the inter-laboratory validation of optimized exposure conditions to ensure robust transferability. The successful application of this integrated protocol to clinically relevant nano-silver burn patch extracts further demonstrates its practical utility, thereby bridging the critical gap between theoretical guidelines and routine nanomaterial safety assessment emphasized in the recent literature [13]. Serum, albumin, and S9 metabolic activation systems present in culture medium may alter nanoparticle size distribution and zeta potential, thereby impeding cellular uptake. Moreover, metallic nanomaterials may release ions that react with dispersion media, generating confounding artifacts. Notably, under S9 metabolic activation conditions, the reaction of silver ions released from Ag40 with chloride-containing media and S9 fractions resulted in silver chloride precipitates, leading to higher apparent intracellular silver content that interferes with accurate result interpretation. Landsiedel et al. [37] demonstrated that proteins within S9 fractions may form protein coronas with nanomaterials; however, the effects on cellular uptake are not uniformly inhibitory and vary depending on nanomaterial type. According to the 2021 European Food Safety Authority (EFSA) guidelines, most insoluble inorganic nanomaterials are not metabolized by conventional enzymatic pathways, and extracellular S9 fractions may interfere with assay performance by reducing nanomaterial bioavailability. Nevertheless, inorganic nanomaterials surface-modified with organic functional groups may exhibit genotoxic effects in the presence of metabolic activation systems. Therefore, the inclusion of S9 fractions should be evaluated on a case-by-case basis [38,39].

CytoB inhibits actin polymerization, thereby blocking phagocytosis and macropinocytosis—pathways essential for nanoparticle internalization. The authors of [40] previously demonstrated that CytoB-induced cytoskeletal damage significantly impairs nanoparticle uptake, leading to false-negative genotoxicity results when CytoB is administered simultaneously with nanomaterials. Consistent with these findings, the present results demonstrated that CytoB significantly inhibited the cellular uptake of Ag40. Consequently, delayed CytoB addition is recommended to avoid disruption of nanomaterial internalization kinetics. While the present study adopted a delayed CytoB addition strategy based on previous reports demonstrating its rationale, a direct comparative evaluation between simultaneous and delayed CytoB addition under the same experimental conditions was beyond the scope of this work. Future studies may further quantify the extent to which this modification enhances detection sensitivity.

Qualitative and quantitative assessment of cellular association revealed higher intracellular silver content in CHL cells compared with TK6 cells, a disparity attributed to the enhanced adsorption of silver nanoparticles on adherent cell surfaces. These findings indicate that distinct cell lines exhibit differential sensitivities in Ag40 quantification. According to the inter-laboratory comparison results documented in OECD TG 359, TK6 cells demonstrated the highest sensitivity for detecting nanomaterial-induced mutagenic effects. Furthermore, marked differences in detection sensitivity were observed among cell lines with varying p53 functional statuses, underscoring the critical importance of intact p53 function in genotoxicity assays [41].

Inter-laboratory validation demonstrated fundamentally concordant results between the two participating laboratories, despite minor variations in baseline values. Under the established test conditions, Ag40 at concentrations of 5–20 µg/mL (nanoscale positive control) induced a dose-dependent increase in micronucleus frequency and yielded consistently positive results. In contrast, polystyrene nanospheres (nanoscale negative control) did not induce significant chromosomal damage and produced stable negative results. These findings collectively confirm that the optimized CBMN assay ensures adequate nanomaterial cellular association and reliably detects Ag40-induced chromosomal breakage [42].

Nano-silver burn patches, widely employed in clinical practice for the management of mild-to-moderate burn wounds and superficial skin injuries with silver nanoparticle loadings ranging from 0.28 to 0.7 g per square meter, were evaluated using the optimized CBMN assay. Leveraging the intrinsic antimicrobial and anti-inflammatory properties of silver nanoparticles, these dressings maintain a moist wound microenvironment, facilitate healing, and reduce the risk of pain and secondary infection. The results demonstrated that dressing extracts at dilution ratios of 25–100% satisfied the cytotoxicity control criteria. No statistically significant elevation in micronucleus frequency or dose-dependent genotoxic trends was observed in any treatment group, yielding unequivocally negative results that indicate that the tested dressings pose no discernible risk of chromosomal damage. Furthermore, cellular silver association analysis revealed extremely low intracellular silver levels, below the instrument detection limit, providing an additional mechanistic explanation for the absence of micronucleus induction.

The optimized assay protocol established in the present study was designed in alignment with the combined framework of OECD TG 487 (2023) and Series on Testing and Assessment No. 359. The rationale for these methodological adaptations was corroborated by evidence from previous studies on diverse nanomaterial classes [43]. Discrepancies in carbon nanotube (CNT) genotoxicity data have been primarily ascribed to the lack of standardization in material dispersion, purity, and exposure conditions [44]. Multi-walled carbon nanotubes (MWCNTs) were demonstrated to induce a dose-dependent rise in micronucleus frequency within acceptable cytotoxicity ranges under nano-optimized conditions, whereas single-walled carbon nanotubes (SWCNTs) posed chromosomal damage risks only at elevated doses [43]. Similarly, neutral iron oxide nanoparticles exhibited negative micronucleus results in single-cell models; however, their toxic effects were conditional, as positively surface-modified iron oxide nanoparticles or those co-cultured with immune cells could indirectly trigger chromosome damage via inflammatory response activation [42], underscoring the necessity of an optimized approach to accurately reflect the toxic characteristics of nanomaterials in in vitro microenvironments.

Furthermore, conventionally used nano-hydroxyapatite with regular particle sizes has been reported to exhibit excellent biocompatibility and no detectable genotoxicity [24]; however, elevated micronucleus frequency was previously observed at extremely high concentrations exceeding 150 ppm, highlighting that the strict concentration and cytotoxicity restrictions imposed by the optimized protocol in the present study are essential to avoid false-positive outcomes from excessive dose escalation. These findings collectively corroborate the rationale underlying the methodological optimizations implemented in this study.

Notwithstanding these advances, the validation of nanomaterial genotoxicity testing methods remains incomplete, as several critical deficiencies persist: the absence of an established particulate positive control, substantial inter-cell-line sensitivity variations, non-standardized CytoB treatment protocols, inadequate linkage between physicochemical characterization and test conditions, and a lack of validated in vitro–in vivo correlation. These limitations were corroborated by the 2024 European Food Safety Authority assessment, which underscored the necessity for further methodological refinement in nanomaterial genotoxicity evaluation.

The present study has several limitations. Validation was performed exclusively with silver-containing nanomaterials. Silver nanomaterials induce genotoxicity primarily through an oxidation-dependent ion release mechanism, wherein Ag^+^ released from AgNPs in oxygen-containing biological environments triggers oxidative stress via glutathione depletion and mitochondrial dysfunction, leading to reactive oxygen species-mediated DNA damage [44,45]. This mechanism, characterized by pronounced particle size dependence, has been corroborated by multiple in vitro and in vivo studies [25]. The applicability of the established method to other commonly used nanomaterials in the biomedical field, such as carbon nanotubes, iron oxide nanoparticles, and nano-hydroxyapatite, remains to be extensively verified in subsequent studies.

Moreover, conventional two-dimensional (2D) cell culture systems are inherently limited by nanoparticle sedimentation, aggregation, and heterogeneous cellular association, and fail to accurately replicate in vivo exposure conditions [46,47]. Time-resolved DLS monitoring of the dosing suspension throughout the exposure period (e.g., at 0, 4, 24, 48, and 72 h) would provide direct evidence of the temporal evolution of hydrodynamic diameter and polydispersity, enabling quantitative discrimination between gradual gravitational sedimentation and true particle aggregation in the culture medium. Compared with 2D culture, three-dimensional (3D) models are capable of faithfully simulating tissue architecture, cellular interactions, and the in vivo microenvironment, while fully preserving cellular morphology and biological characteristics. Nutrient and metabolite exchange in 3D models is consistent with that in living tissue, enabling precise recapitulation of pathological states and enhancing predictive value [48,49]. Furthermore, 3D models have been demonstrated to reduce nanoparticle sedimentation and concentration heterogeneity, and facilitate more uniform particle distribution.

Integration with microfluidic technology has additionally been shown to more effectively restore organ physiological states, establishing 3D culture as a more reliable in vitro system for evaluating nanoparticle association and toxicity [50,51]. Accordingly, future studies should prioritize the optimization of nanomaterial extraction protocols and the implementation of 3D cell culture systems to more accurately simulate in vivo biological behaviors [52].

## 5. Conclusions

We optimized the in vitro cytokinesis-block micronucleus (CBMN) assay using TK6 and CHL cells, which enables effective evaluation of the chromosomal damage risk induced by silver-containing nanomaterials. We expect that this method could be similarly applicable to assessing clastogenic damage induced by other metallic nanomaterials. Based on the methodology developed in the present study, we proposed and developed the Chinese national industry standard YY/T 1897-2023 “Biological evaluation of nanomedical devices—Genotoxicity testing—In vitro micronucleus test in mammalian cells”, which outlines the fundamental concepts described in the present study.

## Data Availability

The original contributions presented in this study are included in the article. Further inquiries can be directed to the corresponding author.
